# Experiencing sexuality in the perinatal period: what are the challenges?

**DOI:** 10.1192/j.eurpsy.2025.2300

**Published:** 2025-08-26

**Authors:** M. M. Figueiredo, R. Pereira, V. Vila Nova

**Affiliations:** 1 Centro Hospitalar Setúbal, ULS Arrábida; 2 USF Santiago de Palmela, Setúbal, Portugal

## Abstract

**Introduction:**

The perinatal period, encompassing pregnancy and the first postpartum year, is a transformative phase in a woman’s life. It involves profound physical, psychological, social, and emotional changes that can significantly impact sexual experiences.

**Objectives:**

The purpose of this work is to address the challenges of experiencing sexuality during the perinatal period.

**Methods:**

Evidence-based review, through research conducted on PubMed and selection of the most relevant studies, published in the last decade, using the keywords: “Sexuality” and “Perinatal period”.

**Results:**

Hormonal fluctuations, physical discomfort, and body image changes during pregnancy can significantly affect sexual desire and satisfaction. By the third trimester, between 83 to 100% of first-time mothers report a decrease in sexual activity. Recent studies indicate that this trend continues into the first postpartum year, with over 60% reporting decreased sexual activity. Postpartum challenges, such as physical recovery, breastfeeding, and fatigue further complicate sexual intimacy. The anticipation of parenthood and shifts in relationship dynamics also play a significant role. Societal pressures and cultural norms regarding sexuality in the perinatal period can influence personal experiences and expectations, and contribute to communication barriers between partners regarding sexual needs and concerns.

**Conclusions:**

The perinatal period presents unique challenges to sexual health and intimacy. A holistic perinatal care, incorporating comprehensive sexual health education and promoting open communication between partners are crucial for addressing these challenges effectively.

**Disclosure of Interest:**

None Declared

